# Behavioral, cognitive and emotional determinants of getting vaccinated for COVID-19 and the mediating role of institutional trust among young adults in Cyprus

**DOI:** 10.1186/s12889-024-19859-y

**Published:** 2024-08-28

**Authors:** Pinelopi Konstantinou, Maria Kyprianidou, Andria Christodoulou, Louise McHugh, Marios Constantinou, Eleni Epiphaniou, Nigel Vahey, Christiana Nicolaou, Nicos Middleton, Maria Karekla, Angelos P. Kassianos

**Affiliations:** 1 Department of Psychology, University of Cyprus, Nicosia, Cyprus; 2 Department of Psychology, School of Sciences, University of Central Lancashire, Larnaca, Cyprus; 3 Department of Social and Behavioral Science, European University Cyprus, Nicosia, Cyprus; 4 Department of Psychology, University College Dublin, Dublin, Ireland; 5 Department of Social Sciences, University of Nicosia, Nicosia, Cyprus; 6 Department of Psychology, Technological University Dublin, Dublin, Ireland; 7 Department of Nursing, Cyprus University of Technology, Limassol, 3041 Cyprus; 8 Department of Applied Health Research, UCL, London, UK

**Keywords:** COVID-19, Vaccination, Health Belief Model, COM-B model, Acceptance and Commitment Therapy

## Abstract

**Background:**

Vaccination uptake is a complex behavior, influenced by numerous factors. Behavioral science theories are commonly used to explain the psychosocial determinants of an individual’s health behavior. This study examined the behavioural, cognitive, and emotional determinants of COVID-19 vaccination intention based on well-established theoretical models: Acceptance and Commitment Therapy (ACT), Capability, Opportunity, Motivation, and Behaviour (COM-B) and the Health Belief Model (HBM). Additionally, it examined the mediating role of institutional trust in the relationship between determinants of these models and vaccination intentions.

**Methods:**

A cross-sectional study was conducted from January to May 2022, where university students in Cyprus completed an online survey.

**Results:**

A total of 484 university students completed the online survey, with 23.8% reporting being vaccinated with fewer than three vaccination doses and/or no intention to vaccinate further. Hierarchical logistic regression analysis showed that higher scores in institutional trust, perceived severity, motivation, physical and psychological capability were significantly associated with higher odds of intending to vaccinate. Higher psychological flexibility and not being infected with COVID-19 were also associated with higher odds of vaccination intention, but not in the final model when all determinants were included. Additionally, significant indirect effects of psychological and physical capability, motivation and perceived severity on vaccination intention were found to be mediated by institutional trust.

**Conclusions:**

When tackling COVID-19 vaccination hesitancy, behavioural, cognitive, and emotional aspects should be considered. Stakeholders and policymakers are advised to implement targeted vaccination programs in young people while at the same time building trust and improving their capabilities and motivation towards getting vaccinated.

**Supplementary Information:**

The online version contains supplementary material available at 10.1186/s12889-024-19859-y.

## Background

Once the coronavirus disease (COVID-19) was announced by the World Health Organization [1] as a global pandemic, lockdowns, and control measures (e.g., mandatory social distancing and mask wearing) were taken by governments worldwide to reduce the impact of COVID-19. Yet, the most effective method for combating the COVID-19 pandemic was vaccination [2–4]. In order to control COVID-19 and prevent future outbreaks, around 70% of citizens in each country had to be vaccinated [4, 5]. Several types of vaccines were developed at the time, demonstrating safety and high effectiveness in preventing COVID-19 infection, hospitalizations, and deaths [2, 4, 6]. However, many people were hesitant to vaccinate with the main reasons at the time being distrust in pharmaceutical companies, and concerns for safety and their efficacy [3, 6, 7]. Additionally, young adults, demonstrated higher levels of hesitancy to COVID-19 vaccinations compared to older adults [8–10]. Although young adults were often considered to be at lower risk to severe outcomes of COVID-19, they play a significant role in COVID-19 transmission and community spread due to their high engagement in social activities [10]. Understanding thus the determinants of vaccination acceptance in this age group is crucial for designing targeted public health strategies that address their specific concerns and barriers to vaccination.

Behavioural science theories such as the Health Belief Model (HBM) [11] and the Capability, Opportunity, Motivation, and Behaviour (COM-B) model [12] are commonly used as frameworks for understanding the factors that influence decision-making by determining what motivates or discourages individuals to engage in health behaviours [13, 14]. The HBM model posits that a health-related behaviour is determined by an individuals’ perceived susceptibility to and severity of a disease, the benefits and barriers of changing a behaviour, and any cues to action [2, 15, 16]. The COM-B model, supports that a behaviour will occur only when the individual has the physical (e.g., being able to travel to vaccination centers) and psychological capability (e.g., understanding the importance of COVID-19 vaccinations) and opportunity to engage in the behaviour and is more motivated to enact that behaviour than any other [17–20]. To our knowledge, no study currently exists examining determinants from both the HBM and COM-B models for COVID-19 vaccination uptake in young adults. Studies examining the HBM [15, 21–25] and COM-B [26] models in the general adult population for COVID-19 vaccination uptake found that higher perceived severity, susceptibility, benefits, cues to action, capability, opportunity and motivation, and lower perceived barriers predicted greater COVID-19 vaccine uptake.

Trust in state authorities was also consistently reported in the general adult population as one of the most highly correlated factors with COVID-19 vaccination uptake and with distrust as one of the main drivers of vaccine hesitancy [4, 6, 8, 23, 27–31]. Individuals who reported higher levels of trust in state authorities were more likely to be vaccinated or intended to get vaccinated against COVID-19 whereas those who did not trust state authorities were more likely to be unvaccinated or demonstrate hesitancy. A global survey examining COVID-19 vaccination acceptance [32] revealed that in countries such as China, South Korea and Singapore in which individuals showed higher trust in state authorities, vaccination acceptance rates tended to exceed 80%. During a global health crisis like the COVID-19 pandemic, marked by widespread misinformation and uncertainty, the pivotal role of the population’s trust in state authorities becomes evident in ensuring successful vaccination campaigns.

Vaccination intention and uptake can be also associated with emotional determinants [33]. Specifically, psychological flexibility is one of the factors that may be related with COVID-19 vaccination intention, with greater psychological flexibility found to be associated with reduced COVID-19 mental health difficulties, better coping with COVID-19 distress and thus higher intention to vaccinate [34–36]. Psychological flexibility is the underlying mechanism of Acceptance and Commitment Therapy (ACT) [37], referring to the ability of fully contacting the present moment as a conscious person by being open and aware of the internal experiences (thoughts, feelings, sensations), and behave based on associated values [38–40]. ACT aims to improve psychological flexibility through improvement in its six core processes of change, namely acceptance, defusion, self-as-context, present moment awareness, values and committed action [39].

In the long-term, WHO emphasized that COVID-19 remains a global health threat [41]. Therefore, it is important to understand the determinants and mechanisms that drive young adults toward COVID-19 vaccinations to effectively control new variants of COVID-19 and to possibly mitigate the severity and progress of future pandemic outbreaks. The present study aims to identify the behavioural, cognitive, and emotional factors that can drive young adults to vaccinate or towards intending to vaccinate against COVID-19 using components from the ACT, COM-B, and HBM models. In addition, it aims to examine the mediating role of institutional trust in the relationship between determinants from these models with vaccination intention and uptake.

## Methods

### Research design

A cross-sectional study was conducted from January to May 2022, where university students in Cyprus completed an online survey. During this period, in Cyprus, the Omicron variant was spreading with the highest infection rates observed throughout the whole COVID-19 pandemic [42]. The third (booster) vaccine dose for COVID-19 was available to all residents of Cyprus and protective measures were in place including mandatory mask wearing. The vaccination rollout followed a phased approach, with priority groups receiving vaccinations first (e.g., healthcare workers, elderly, high-risk populations). Vaccinations were offered free of charge to all residents in Cyprus and were strongly recommended by the government with campaigns delivered in the social media. Individuals needed to demonstrate a mandatory “SafePass” for most activities including going out for entertainment, by showing proof either of being vaccinated with the first dose as administrated at least three weeks prior, or a negative rapid or PCR test not older than 72 h or recovery from COVID-19 in the past six months.

### Participants and procedures

Eligibility criteria for participation included being a university student in Cyprus, aged older than 18 and with adequate understanding of Greek language. A convenience sampling approach was used. The study was advertised through email lists in four universities (Cyprus University of Technology, European University of Cyprus, University of Cyprus, and University of Nicosia). Three universities were in Nicosia and one in Limassol. Power sample calculation using linear multiple regression indicated that a sample size of at least 74 individuals could provide a medium effect size and high power for the vaccination intention outcome (*Cohens f*^*2*^ = 0.15, *Power* = 0.95, *p* < .05; [43]. Students who completed the survey took part in a draw to receive gift vouchers as incentives. The RedCap software (https://redcap.ucy.ac.cy/) was used to collect the data. Prior to completing the survey, participants provided informed consent electronically. The average duration of survey completion was ten minutes. The study was approved by the Cyprus National Bioethics Committee (reference: ΕΕΒΚ ΕΠ 2019.01.131).

### Measures

#### Outcome

##### Vaccination intention

Participants were asked whether they have been vaccinated with the COVID-19 vaccine and if not, whether they plan to get vaccinated (Supplementary Material A). Participants who reported vaccination, were asked to provide further clarifications, namely if they have been vaccinated with the second and third/booster dose, and the specific type of vaccine.

#### Determinants

The measures that were not available in Greek language (i.e., HBM questionnaire, COM-B model questionnaire) were translated following standard forward and backward translation procedures [44]. All measures can be found in Supplementary Material A.

##### Emotional determinants

Psychological Flexibility was assessed with the Psy-Flex scale [45, 46]. The Greek version was used [46], comprised of 10-items rated on a 5-point Likert scale (1 = very seldom to 5 = very often). A score is calculated by summing up all items, with higher scores indicating higher psychological flexibility. The Psy-Flex demonstrated good psychometric properties (convergent and divergent validity, internal consistency: Raykov estimation range 0.78–0.97) in both English and Greek versions [45, 46]. In this study, Psy-Flex demonstrated good internal consistency (α = 0.82).

##### Cognitive determinants

Cognitive determinants were assessed via a questionnaire [47] developed for patients and based on two components of the HBM [11] and the Cognitive Fusion Questionnaire (CFQ) [48, 49]. The HBM questionnaire is comprised of six items, divided into two subscales, rated on a 6-point Likert scale (1 = absolutely disagree to 6 = absolutely agree). A score was calculated by summing up items for each subscale with higher scores on each sub-scale indicating greater perceived susceptibility and perceived severity respectively. Perceived susceptibility refers to individuals’ perception of likelihood to contract COVID-19, whereas perceived severity refers to their perception of the severity and the consequences of contracting COVID-19. The HBM questionnaire has shown good psychometric properties with satisfactory reliability and validity (α = 0.70 to 0.85) [47]. In this study, both subscales demonstrated acceptable internal consistency (α = 0.70).

The Greek version of CFQ [49] was used to assess cognitive fusion. Cognitive fusion refers to the excessive control or fusion of people to their thoughts and its one of the processes of change included in ACT [48]. It is comprised of seven items rated on a 7-point Likert scale (1 = never true to 7 = always true) yielding a total score, with higher scores indicating greater cognitive fusion. CFQ has shown good psychometric properties with excellent internal consistency (α = 0.88 to 0.96) and good convergent validity [48, 49]. In this study, CFQ demonstrated excellent internal consistency (α = 0.94).

##### Behavioral Determinants

Behavioral determinants were assessed using the COM-B model questionnaire [50] and the Valuing Questionnaire (VQ) [51, 52]. It is important to note that the COM-B model is not solely focused on behavior; it provides a comprehensive framework for understanding the interplay of capabilities, opportunities, and motivations that influence behavior. The COM-B questionnaire was used to assess physical and psychological capabilities, social and physical opportunities, and both reflective and automatic motivations to provide a nuanced understanding of behavioral determinants. Capability referred to the physical (e.g., endurance or reliance to undergo the COVID-19 vaccination process) and psychological capability (e.g., understanding the importance of COVID-19 vaccines, decision-making skills on getting vaccinated, recognizing potential side effects) to vaccinate. Opportunity referred to the physical and social environment that is outside of the individual and could support vaccination. Motivation referred to both reflective (e.g., conscious decision making) and automatic processes (e.g., emotions) that guided and directed the decision to vaccinate. It is comprised by 6-items rated on a 10-point Likert scale ranging from 0 (strongly disagree) to 10 (strongly agree). A score is calculated on each item with higher scores indicating greater physical and social opportunities, motivation, and physical and psychological capabilities. COM-B showed good psychometric properties with good test-retest reliability and discriminant validity [50].

The Greek version of VQ [52] was used to assess two aspects related to values progress and obstruction. It is comprised of 10 items divided into two subscales (Progress and Obstruction), rated on a 7-point Likert scale (0 = Not at all true to 6 = Completely true).Values progress refers to individuals’ awareness of what is important and living in accordance to their values whereas values obstruction refers to disruption of valued living due to deviation from values and avoidance of negative internal experiences [51]. A total score is calculated for each subscale and higher scores on each sub-scale indicate greater progress or obstruction toward valued living during the past week. VQ showed good psychometric properties with good internal consistency for both subscales (α = 0.74 to 0.89) and convergent validity [51, 52]. In this study, VQ demonstrated good internal consistency (α = 0.82 for both subscales).

##### Socio-demographic information

Socio-demographic information included age (in years), gender (female/male/other), study programme (health sciences vs. all other sciences), having under-aged children (yes/no) and living situation (living alone/living with parents, own family, or roommates).

##### COVID-19 infection

Participants responded to a question on whether they had been infected with COVID-19 (Yes/No/Don’t Know).

##### Institutional trust

Institutional trust was assessed using one item of trust towards state authorities, scored on a 7-point Likert scale ranging from 1 (very little trust) to 7 (a lot of trust).

### Statistical analyses

The determinants of vaccination between participants being unvaccinated, being vaccinated with at least one dose but with no intention and vaccinated with at least one dose but with an intention to vaccinate were firstly compared using one-way ANOVAs. Due to the non-significant differences in any of the determinants examined (Supplementary Material B) between participants being unvaccinated and those being vaccinated with at least one dose but with no intention to receive the next dose available, these two categories were combined to increase the statistical power in the main analyses. Therefore, *vaccination intention* was coded as a binary variable: (a) fully vaccinated with all three doses or if vaccinated with less with an intention to vaccinate, and (b) unvaccinated or vaccinated with less than three doses with no intention to receive the next dose available.

Participants’ socio-demographic characteristics were presented using means and standard deviations (SD) for continuous variables, and absolute (n) and relative (%) frequencies for categorical variables. Assumption of normality was inspected for continuous variables statistically using the Kolmogorov-Smirnov test and skewness and kurtosis values. Normality was found as met for all variables, thus correlations between the continuous determinants were examined with Pearson’s r correlations, whereas correlations between categorical determinants were examined using chi-square test. Independent samples t-tests were used to compare the continuous determinants between the two groups according to their vaccination intentions.

Hierarchical logistic regression models were conducted to examine the association between the determinants and vaccination intentions after adjusting for age, gender, study programme, having under-aged children, and living situation. First, the COVID-19 infection variable was added (Model 1), followed by institutional trust (Model 2), emotional factors (psychological flexibility; Model 3), cognitive factors (perceived susceptibility, perceived severity, cognitive fusion; Model 4), and finally behavioral factors (physical and social opportunity, motivation, physical and psychological capability, values obstruction, values progress; Model 5). The reason of adding the determinants in this order was to examine first the inner factors of vaccination intention and then the factors related to the behavior of participants.

Mediation models were then conducted using PROCESS macro for SPSS [53] to examine whether institutional trust mediated the relationship between the factors found to be significantly associated with vaccination intentions in the hierarchical logistic regression models. The bootstrapping mediation method was used with 5000 resampling. All statistical tests performed were two-sided with the statistical significance level set at α = 0.05. The SPSS software (Version 25.0) was used to conduct all statistical analyses.

## Results

### Participants characteristics

The sample consisted of 484 students from two public (*n* = 249, 51.4%) and two private (*n* = 235, 48.6%) universities in Cyprus (see Table 1). Most participants were females (*n =* 392, 81.0%), with mean age 25.7 (*SD* = 7.5, *range* = 18 to 58), living with another person (*n =* 378, 78.1%), and without under-aged children (*n* = 434, 89.7%). Only 33 participants were registered in health-related programmes (6.8%). Most participants (*n* = 302, 62.4%) reported that they had not or did not know if they had contracted COVID-19. In terms of the primary outcome, 369 participants (76.2%) were fully vaccinated with all three doses or less but with intention to vaccinate whereas 115 (23.8%) reported being unvaccinated or vaccinated with less than three doses and had no intention to receive the next dose. Of the latter group, 88 participants (18.2% of the total sample) reported that they were not vaccinated at all, with no vaccination intention.

**Table 1:** Characteristics of the participants and means and associations between determinants with vaccination intention outcome

Characteristics	Overall (*n* = 484)	Vaccination Intention		*p*-value
		Fully vaccinated with all 3 doses/less but with intention to vaccinate (*n* = 369)	Less than 3 doses & no intention to vaccinate (*n* = 115)	
Mean Age (SD)	25.7 (7.6)	25.8 (7.5)	25.4 (7.8)	0.65^†^
Gender, *n* (%)				0.78^‡^
Female	392 (81.0)	297 (80.5)	95 (82.6)	
Male	89 (18.4)	70 (19.0)	19 (16.5)	
Other	3 (0.6)	2 (0.5)	1 (0.9)	
University type, *n* (%)				0.21^‡^
Public	249 (51.4)	184 (49.9)	65 (56.5)	
Private	235 (48.6)	185 (50.1)	50 (43.5)	
Specific university, *n* (%)				0.10^‡^
University of Cyprus	221 (45.7)	168 (45.5)	53 (46.1)	
University of Nicosia	115 (23.8)	93 (25.2)	22 (19.1)	
European University of Cyprus	97 (20.0)	76 (20.6)	12 (10.4)	
Cyprus University of Technology	28 (5.8)	16 (4.3)	21 (18.3)	
Other	23 (4.8)	16 (4.3)	7 (6.1)	
Health Sciences students, *n* (%)	33 (6.8)	30 (8.1)	3 (2.6)	**0.04** ^‡^
Having under aged children, *n* (%)	50 (10.3)	40 (10.8)	10 (8.7)	0.51^‡^
Living situation, *n* (%)				0.18^‡^
Living alone	106 (21.9)	86 (23.3)	20 (17.4)	
Living with another person (parents/own family/ roommates)	378 (78.1)	283 (76.7)	95 (82.6)	
COVID-19 infection, *n* (%)				**< 0.001** ^‡^
Yes	182 (37.6)	121 (32.8)	61 (53.0)	
No/ Don’t know	302 (62.4)	248 (67.2)	54 (47.0)	
Institutional Trust	3.4 (1.8)	3.99 (1.60)	1.70 (1.15)	**< 0.001** ^†^
Emotional Factors				
Psychological Flexibility	21.7 (5.9)	22.26 (5.78)	19.90 (5.95)	**< 0.001** ^†^
Cognitive Factors				
Cognitive Fusion	26.5 (10.6)	27.32 (10.30)	23.90 (11.25)	**0.01** ^†^
Perceived susceptibility	9.9 (3.8)	10.58 (3.43)	7.56 (3.85)	**< 0.001** ^†^
Perceived severity	10.0 (3.3)	10.71 (3.04)	7.56 (3.18)	**< 0.001** ^†^
Behavioral Factors				
Values progress	19.7 (5.9)	19.63 (5.56)	20.08 (6.71)	0.47^†^
Values obstruction	12.2 (6.6)	12.65 (6.43)	10.90 (6.91)	**0.01** ^†^
Physical Opportunity	8.8 (2.3)	9.20 (1.62)	7.50 (3.34)	**< 0.001** ^†^
Social Opportunity	8.2 (2.6)	8.85 (1.86)	6.22 (3.51)	**< 0.001** ^†^
Motivation	7.2 (3.6)	8.76 (2.13)	2.29 (2.92)	**< 0.001** ^†^
Physical Capability	9.0 (2.3)	9.66 (0.95)	7.02 (3.80)	**< 0.001** ^†^
Psychological Capability	7.5 (3.4)	8.74 (2.06)	3.37 (3.69)	**< 0.001** ^†^

Note. Bold values indicate statistically significant associations

^†^Differences between vaccination intention groups were tested using independent samples t-test

^‡^Differences between vaccination intention groups were tested using chi-square test

### Determinants of COVID-19 vaccination intention

Bivariate correlations between continuous determinants are available in Supplementary Material C, with most of them being moderately or slightly correlated. With respect to the association between categorical determinants with the vaccination intention outcome (Table 1), young adults who were unvaccinated or had no intention to vaccinate further, were more likely to having been infected with COVID-19. Regarding continuous determinants associated with the vaccination intention outcome, young adults who were unvaccinated or had no intention to vaccinate further, reported lower trust in state authorities, psychological flexibility, cognitive fusion, perceived susceptibility and severity, values obstruction, physical and social opportunity, motivation, and physical and psychological capability to vaccinate than those who were fully vaccinated with all doses or had intention to vaccinate further (Table 1).

After adjusting for the socio-demographic variables, in the final model, institutional trust (*OR =* 1.67, *95% CI*: 1.22, 2.28), perceived severity of COVID-19 (*OR =* 1.23, *95% CI*: 1.04, 1.45) and the behavioral factors from the COM-B model of greater motivation (*OR =* 1.53, *95% CI*: 1.31, 1.79), physical capability (*OR =* 1.38, *95% CI*: 1.09, 1.74) and psychological capability (*OR =* 1.24, *95% CI*: 1.06, 1.45) were associated with a higher probability to be fully vaccinated or intending to get vaccinated (Table 2). Institutional trust was a consistently statistically significant predictor of vaccination intention in all models, with higher trust associated with up to three times higher likelihood to be vaccinated. Institutional trust did not attenuate much after adding cognitive and emotional factors in the model but attenuated only when behavioral factors were added, although it remained statistically significant. COVID-19 infection status was also a consistently statistically significant predictor of vaccination intention in all models except of the final one when the behavioral factors were added in the model. Specifically, young adults who were not infected or did not know whether they were infected with COVID-19 were more likely to be unvaccinated or not intending to vaccinate further (Table 2). Additionally, perceived severity was a consistent significant predictor of vaccination intention, with similar odds showed in the final model when the behavioral factors were added. Psychological flexibility was a significant predictor of vaccination intention only in Model 3 (*OR* = 1.11, *95% CI*: 1.06, 1.17), with those having higher psychological flexibility being associated with a higher probability of being fully vaccinated or intending to get vaccinated. However, when the cognitive and behavioral factors were added in the model, this association attenuated and lost statistical significance. Cognitive fusion, perceived susceptibility, values progress and obstruction, and physical and social opportunity were not significant determinants of vaccination intention in any of the Models (Table 2).

**Table 2:** Results from hierarchical logistic regression on vaccination intention

Characteristics	Model 1^†^	Model 2^‡^	Model 3^§^	Model 4^¶^	Model 5^††^
COVID-19 infection					
No/Don’t Know	*Ref*	*Ref*	*Ref*	*Ref*	*Ref*
Yes	**0.41 (0.26**,** 0.63)**	**0.38 (0.22**,** 0.67)**	**0.36 (0.20**,** 0.65)**	**0.42 (0.22**,** 0.79)**	0.53 (0.23, 1.22)
Institutional Trust	**-**	**2.99 (2.41**,** 3.73)**	**3.05 (2.44**,** 3.80)**	**2.78 (2.21**,** 3.51)**	**1.67 (1.22**,** 2.28)**
Emotional Factors					
Psychological Flexibility	**-**	**-**	**1.11 (1.06**,** 1.17)**	1.06 (1.00, 1.12)	1.05 (0.96, 1.15)
Cognitive Factors					
Cognitive Fusion	**-**	**-**	**-**	1.03 (1.00, 1.06)	1.05 (0.99, 1.11)
Perceived Susceptibility	**-**	**-**	**-**	1.00 (0.89, 1.13)	0.94 (0.81, 1.10)
Perceived Severity	**-**	**-**	**-**	**1.23 (1.07**,** 1.41)**	**1.23 (1.04**,** 1.45)**
Behavioral Factors	**-**				
Values’ Progress	**-**	**-**	**-**	**-**	1.01 (0.94, 1.09)
Values’ Obstruction	**-**	**-**	**-**	**-**	0.95 (0.87, 1.05)
Physical Opportunity	**-**	**-**	**-**	**-**	0.81 (0.63, 1.04)
Social Opportunity	**-**	**-**	**-**	**-**	0.97 (0.76, 1.23)
Motivation	**-**	**-**	**-**	**-**	**1.53 (1.31**,** 1.79)**
Physical Capability	**-**	**-**	**-**	**-**	**1.38 (1.09**,** 1.74)**
Psychological Capability	**-**	**-**	**-**	**-**	**1.24 (1.06**,** 1.45)**

Note. Data are given as Odds Ratio (95% Confidence Intervals), Bold font indicates statistical significance at *p* < .05. ^†^Model 1: COVID-19 infection; ^‡^Model 2: Model 1 determinant & Trust in state authorities; ^§^Model 3: Model 2 determinants & Psychological Flexibility; ^¶^Model 4: Model 3 determinants & Cognitive Factors; ^††^Model 5: Model 4 determinants & Behavioral Factors

### Mediators of Vaccination Intention

**Fig. 1:**
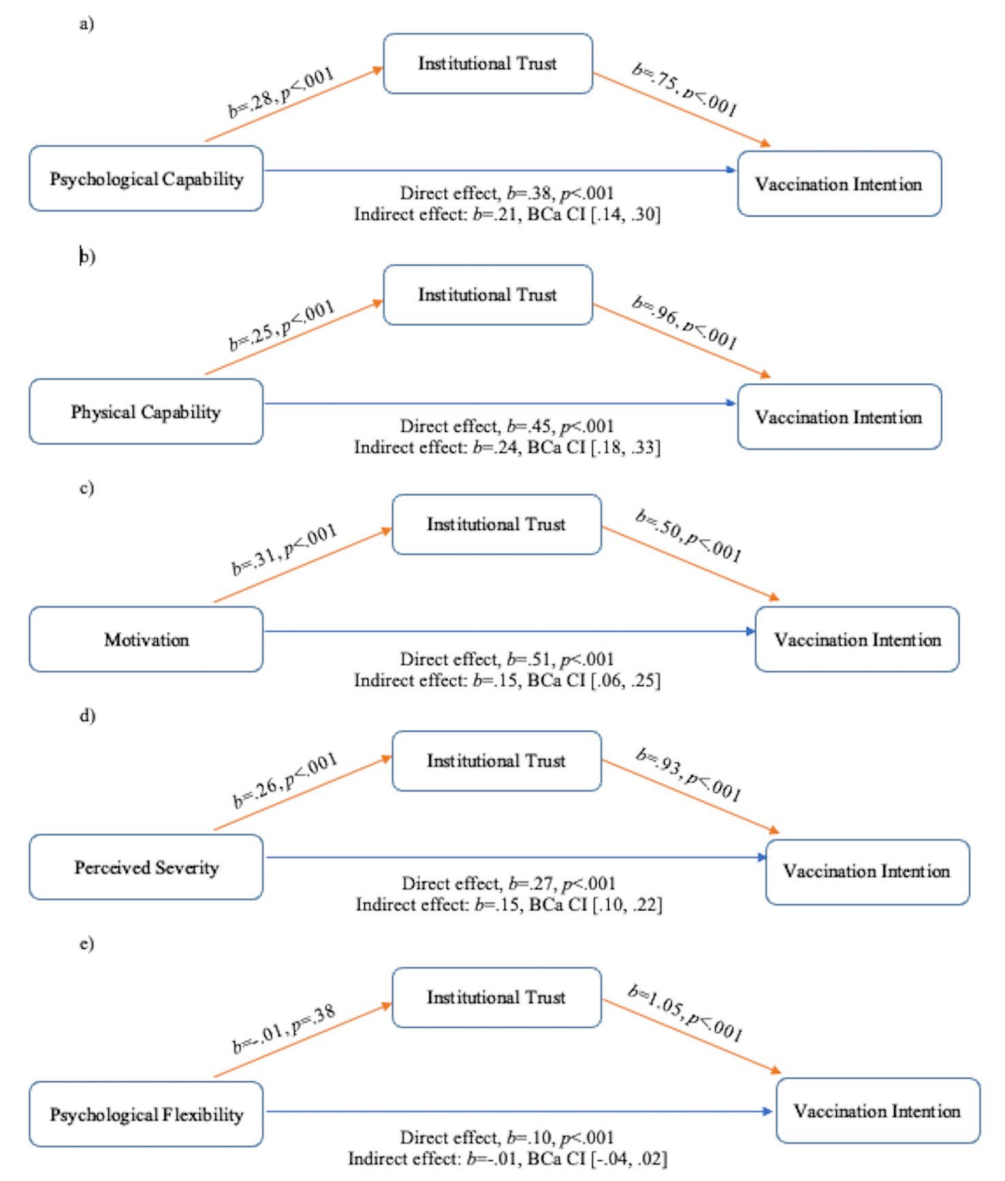
Examination of institutional trust as a mediator between various determinants and vaccination intention

Institutional trust was then examined as a mediator between the determinants of psychological capability, physical capability, motivation, perceived severity, and psychological flexibility with vaccination intention (Fig. 1 a-e). Significant indirect effects of psychological capability (*b* = 0.21, *95% BCa CI* [0.14, 0.30]; Fig. 1a), physical capability (*b* = 0.24, *95% BCa CI* [0.18, 0.33]; Fig. 1b), motivation (*b* = 0.15, *95% BCa CI* [0.06, 0.25]; Fig. 1c) and perceived severity (*b* = 0.15, *95% BCa CI* [0.10, 0.22]; Fig. 1d) on vaccination intention were found to be mediated by institutional trust. However, institutional trust did not mediate the association between psychological flexibility and vaccination intention (*b*=-0.01, *95% BCa CI* [-0.04, 0.02]; Fig. 1e).

## Discussion

In the present study, three out of four university students in Cyprus during the COVID-19 pandemic presented with high intentions to vaccinate with all three doses or with less but intending to get further vaccinated with booster doses. This may be related to the measures adopted by universities in Cyprus at the time as a response to COVID-19, whereby students had to be either vaccinated or having a valid 48-hour rapid test to be allowed to attend the university classes. Even so, one in four (24%) reported that they were unvaccinated or vaccinated with less than three doses with no intention to get further vaccinated, suggesting the importance of providing evidence-based and tailored interventions in younger adults that are designed based on the factors associated with greater vaccination intentions [4, 8, 54].

Examination of the behavioral determinants of the COM-B model showed that young adults who were more motivated to vaccinate and had greater physical and psychological capability were more likely to be fully vaccinated or intending to get vaccinated. It should be noticed that students who received all three doses may differed from other groups because they had already completed with the vaccinations. Combined with previous studies [26, 55, 56], our findings suggest that individuals who are well-informed about COVID-19 vaccines, their safety and importance (e.g., reduce severe illness, death, protect themselves and family), and are optimistic about their effectiveness are more likely to vaccinate. Conversely, physical and social opportunity were not significant determinants. In the case of physical opportunity this is not surprising, since during the study period (January-May 2022), all residents in Cyprus were able to vaccinate as the third (booster) vaccine dose was freely available to everyone. However, concerning social opportunity, which involves the influence of the social environment and cultural norms, the absence of significant results may be attributed to specific contextual factors, such as cultural values and well-developed healthcare infrastructure [19]. For example, in collectivist cultures, where community values are important, individuals are more likely to receive the COVID-19 vaccine than in individualistic cultures [57]. Cultural values might act also as mediators in the relationship between social opportunity and vaccination intention such as specific religious beliefs that encourage or discourage vaccination and trust in government and healthcare systems. It is also possible that social opportunity indirectly affects vaccination intention via the mediating effect of motivation in university students [58]. In contrast, other studies in Iran and Thailand [26, 56] found that physical and social opportunity were the strongest predictors of vaccination acceptance among an adult population. This variation in results might be due to the fact that Cyprus is a small country with less barriers to vaccine access and demonstrate how the contextual and geographical aspects of each population can determine the importance of the COM-B factors on changing health behaviors. Therefore, our findings suggest that although both internal (e.g., motivation and capabilities) and external factors of the individual (e.g., ease of access) can be important, their significance is further influenced by the specific context and cultural norms of the studied population.

When examining the cognitive determinants based on the HBM model, perceived severity was associated with greater likelihood to be fully vaccinated or intending to get vaccinated. Perceived susceptibility was not a significant predictor of vaccination intentions. This aligns with existing evidence [23, 25, 59, 60], which suggests that individuals who perceive that there is a severe health threat and that contracting COVID-19 is serious, are more inclined to take actions toward their health. The non-significant findings of perceived susceptibility could be explained by the fact that a great number of young adults perceived that they face a lower risk of COVID-19 infection and are less likely to experience any symptoms of COVID-19 due to their young age [4, 23].

With respect to the emotional determinants based on the ACT model, we found that psychological flexibility significantly predicted vaccination intention but only in isolation and before considering the HBM and COM-B determinants in the hierarchical model. The models have distinct theoretical underpinnings and offer somewhat different explanations for behavior but not necessarily additive. This may also relate to present study’s findings that the factors of the three theoretical models were moderately or slightly inter-correlated. In addition, the cognitive factors (cognitive fusion) and behavioral factors (values progress and obstruction) were not significant determinants of vaccination intention on their own, yet are encompassed within the overall psychological flexibility construct [39]. Our findings suggest that although being psychologically flexible can somewhat influence intention to vaccinate, other factors related more directly to COVID-19 pandemic and vaccination behavior (i.e., COVID-19 severity, motivation, and capability to vaccinate) appear to be more strongly related to vaccination intentions.

Trust in state authorities also predicted vaccination intention, with young adults who reported greater trust in state authorities being more likely to be fully vaccinated or intending to receive booster doses. Its further examination as a mediator in the relationship between behavioral determinants of psychological and physical capability, motivation, cognitive determinant of perceived severity, and emotional determinant of psychological flexibility with vaccination intentions showed that trust in state authorities significantly mediated all relationships except the one with psychological flexibility. Our results thus underscore the pivotal role of trust in state authorities in influencing and modulating perceptions and motivations that drive vaccination intentions [61]. This aligns with global findings in adult populations, where higher trust in state authorities consistently emerges as a key determinant of COVID-19 vaccination uptake and as one of the main drivers of vaccine hesitancy [4, 6, 8, 23, 27, 28, 62]. Findings of the present study further suggest that the relationship between psychological flexibility and vaccination intention might be therefore possibly influenced by other factors such as individuals’ coping styles and self-efficacy [33]. On the other hand, perceiving COVID-19 as a severe disease, having the physical capabilities and the knowledge, skills, and motivation to vaccinate are not only significant predictors of intentions on their own but are also modulated by the level of trust in state authorities. In particular, the positive impact of psychological and physical capability, motivation, and perceived severity on vaccination intention was facilitated through greater trust in state authorities. It is therefore crucial that recommendations to vaccinate are provided by trusted sources.

### Limitations

The results of this study need to be interpreted considering its limitations. First, data collection was conducted using a convenient sampling approach, promoted through university email lists, and was also conducted online, limiting the study’s representativeness. Moreover, young adults can include other groups as well which do not study at university and future studies can expand this to other groups of young adults as well. However, it was not the intention to provide prevalence estimates of vaccination intention but to explore its association with a range of cognitive, emotional, and behavioral determinants. Our findings can be used as the first step and can inform longitudinal studies with bigger and more representative samples. Secondly, this study included only self-reported vaccination uptake data with three out of four students in Cyprus reporting being fully vaccinated or intending to get vaccinated, suggesting the careful generalization to other contexts and populations. Additionally, due to the cross-sectional design of the study only associations between the variables could be examined and not causal relationships. Moreover, some determinants of vaccination intentions were examined by only one item each (i.e., COM-B factors, trust in state authorities). Future studies are thus suggested to examine these determinants with more robust self-report measures and longitudinally to reach more definite conclusions. In addition, we recognize that the survey was used to collect only some aspects of emotional reactions such as psychological flexibility and future studies can include other aspects such as stress and mood. Finally, the responses of participants who were fully vaccinated might have been ambiguous in reflecting their motivation for vaccination in the COM-B model questionnaire, since they had already completed their vaccination. Future studies examining motivation to vaccination should consider modifying the item (e.g., “If a fourth dose of the COVID-19 vaccine is available, I am motivated to vaccinate”).

### Policy and research implications

Motivation to vaccinate is distinct to intention and actual behavior. While an individual might present with high motivation to vaccinate, may not always translate into concrete intentions to receive the COVID-19 vaccine or proceed into actual vaccination behavior [63]. Findings of the present study supported that vaccination intention was influenced not only by motivation, but also by other determinants such as perceived severity and capabilities of the individuals. Institutional trust was also an important factor explaining the relationship between motivation and vaccination intention. Future studies can explore longitudinally additional mediating factors between motivation, intention and actual vaccination behavior such as cultural values, social norms and vaccination beliefs. Researchers should clearly distinguish and recognize the differences between these three constructs so as to develop a more nuanced understanding of COVID-19 vaccination decision-making.

Vaccination campaigns could be tailored for young adults focusing on improving their motivation and capabilities for engaging in vaccination. For example, capability can be increased by information provision and skills training or by discussing and addressing their worries. Increasing capabilities to vaccinate might also affect the motivation of individuals as it is suggested that these two are inter-related [17, 20, 26]. Strategies such as improving perceived knowledge on vaccination or motivational interviewing (e.g., discuss concerns and enhance motivation to vaccinate) can be adopted during daily education to improve motivation and psychological capabilities [64–67]. Motivated individuals exhibit a greater likelihood of accepting and actively participating in COVID-19 vaccination, with their increased motivation possibly contributing to achieving herd immunity and reducing the transmission of infectious diseases within communities [26]. In addition, motivated young adults are more likely to perceive vaccination important and emphasize its benefits; therefore, suggesting public health campaigns and interventions should focus on improving vaccination importance first and consequently motivation to promote vaccination intentions. Campaigns designed by experts can be also delivered to increase motivation of individuals to vaccinate.

At the same time, campaigns can strive to educate young adults on the consequences and severity of COVID-19 to their health and to that of their significant others. Strategies including providing scientific evidence about COVID-19 symptoms, transmission, and health consequences, emphasizing the potential health risks (e.g., long COVID health effects) can be adopted to enhance awareness on the perceived severity of COVID-19 on individuals’ health [68, 69]. State authorities could introduce vaccine programs that incorporate behavior change techniques such as credible sources [12], while building or consolidating trust to those who are more skeptical by being transparent on processes and decisions taken. Involving people who are more likely to be trusted and implementing stratified vaccination campaigns to tackle youth populations may be instrumental in increasing vaccination uptake [70].

## Conclusions

Increasing vaccination uptake is a complex health problem, as it is related with numerous behavioral, cognitive, and emotional factors. Trust in state authorities, cognitive (perceptions on COVID-19 severity), and behavioral determinants (motivation, physical and psychological capability) were the strongest predictors of vaccination intentions. Trust in state authorities was also found to mediate the relationship of vaccination intentions with psychological capability, motivation, and perceived severity. In addition, psychological flexibility and COVID-19 infection status were significant predictors of vaccination intention when examined in isolation. This study is the first using components from ACT, COM-B and HBM to understand the determinants of COVID-19 vaccination intentions among young adults. To tackle non- or under-immunized young adults more effectively, it is advised that vaccination campaigns should focus on educating them on the consequences that COVID-19 might has on their health while at the same time improving their capabilities and motivation for engaging in vaccination. State authorities should also aim to build trust with younger adults and deliver vaccination campaigns by trusted sources (e.g., scientists).

### Electronic supplementary material

Below is the link to the electronic supplementary material.

Supplementary Material 1

## Data Availability

Data are available upon request.
