# Supplementary material for: Behavioral, cognitive and emotional determinants of getting vaccinated for COVID-19 and the mediating role of institutional trust among young adults in Cyprus
Source: BMC Public Health. 2024 Aug 28;24:2336. doi: 10.1186/s12889-024-19859-y (PMC11351732; doi:10.1186/s12889-024-19859-y)
Supplement: Supplementary file 1 — Supplementary Material 1 [file 12889_2024_19859_MOESM1_ESM.docx]

# Supplementary Materials

## Supplementary Material A

**Measures used in the present study**

1. **Vaccination Intention and Uptake**

Have you been vaccinated with the COVID-19 vaccine?

Yes

No

**If Yes:**

- Why did you get vaccinated? (open-ended)
- When did you receive the last dose? (open-ended)
- Which vaccine did you receive?
  - Pfizer
  - Moderna
  - Astra Zeneca
  - Johnson & Johnson
  - Other
- Did you receive the booster vaccine (3^rd^ dose for Pfizer/Moderna/Astra Zeneca & 2^nd^ dose for Johnson & Johnson)
  - Yes
  - No
- If not, do you plan to receive the booster vaccine?
  - Yes
  - No

**If No:**

- If not, do you plan to get vaccinated for COVID-19?
  - Yes
  - No

1. **Psy-Flex scale**

The questions refer to your experiences in the last seven days.

**Being present**

Even if I am somewhere else with my thoughts I can focus on what’s going on in important moments.

| very often | often | from time to time | seldom | very seldom |
| --- | --- | --- | --- | --- |

(5) (4) (3) (2) (1)

**Being open for experiences**

If need be, I can let unpleasant thoughts and experiences happen without having to get rid of them immediately.

| very often | often | from time to time | seldom | very seldom |
| --- | --- | --- | --- | --- |

(5) (4) (3) (2) (1)

**Leaving thoughts be**

I can look at hindering thoughts from a distance without letting them control me.

| very often | often | from time to time | seldom | very seldom |
| --- | --- | --- | --- | --- |

(5) (4) (3) (2) (1)

**Steady self**

Even if thoughts and experiences are confusing me I can notice something like a steady core inside of me.

| very often | often | from time to time | seldom | very seldom |
| --- | --- | --- | --- | --- |

(5) (4) (3) (2) (1)

**Steady self**

I recognize that myself is not just my thoughts or worries.

| very often | often | from time to time | seldom | very seldom |
| --- | --- | --- | --- | --- |

(5) (4) (3) (2) (1)

**Awareness of one’s own values**

I determine what’s important for me and decide what I want to use my energy for.

| very often | often | from time to time | seldom | very seldom |
| --- | --- | --- | --- | --- |

(5) (4) (3) (2) (1)

**Being engaged**

I engage thoroughly in things that are important, useful, or meaningful to me.

| very often | often | from time to time | seldom | very seldom |
| --- | --- | --- | --- | --- |

(5) (4) (3) (2) (1)

**Being benevolent**

I face others with tolerance, benevolence and compassion.

| very often | often | from time to time | seldom | very seldom |
| --- | --- | --- | --- | --- |

(5) (4) (3) (2) (1)

**Being benevolent**

I face myself with tolerance, benevolence and compassion.

| very often | often | from time to time | seldom | very seldom |
| --- | --- | --- | --- | --- |

(5) (4) (3) (2) (1)

**Being on track**

I’m heading in the right direction in my life.

| very often | often | from time to time | seldom | very seldom |
| --- | --- | --- | --- | --- |

(5) (4) (3) (2) (1

1. **Health Belief Model (HBM) Questionnaire**

| \| 1=Strongly disagree **(SD)** 2=Moderately disagree **(MD)** 3=Slightly disagree **(D)** \| 4=Slightly agree **(A)** 5=Moderately agree **(MA)** 6=Strongly agree **(SA)** \| \| --- \| --- \| |
| --- | --- | --- |

|  | | **SD** | **MD** | **D** | **A** | **MA** | **SA** |
| --- | --- | --- | --- | --- | --- | --- | --- |
| 1 | I have an increased risk of getting infected by COVID-19 | 1 | 2 | 3 | 4 | 5 | 6 |
| 2 | I am concerned about the risk of getting the COVID-19 | 1 | 2 | 3 | 4 | 5 | 6 |
| 3 | I get sick more easily than other people my age | 1 | 2 | 3 | 4 | 5 | 6 |
| 4 | COVID-19 may lead to serious health problems | 1 | 2 | 3 | 4 | 5 | 6 |
| 5 | I am afraid that if I get infected by COVID-19 I will be very sick | 1 | 2 | 3 | 4 | 5 | 6 |
| 6 | My life will change if I get infected by COVID-19 | 1 | 2 | 3 | 4 | 5 | 6 |
| 7 | The benefits for my health from COVID-19 vaccine uptake are high | 1 | 2 | 3 | 4 | 5 | 6 |
| 8 | The difficulties in getting vaccinated with the new COVID-19 vaccine are high | 1 | 2 | 3 | 4 | 5 | 6 |
| 9 | I have the skills (e.g., book an appointment) to get vaccinated against COVID-19. | 1 | 2 | 3 | 4 | 5 | 6 |

1. **Cognitive Fusion Questionnaire (CFQ)**

Below you will find a list of statements. Please rate how true each statement is for you by circling a number next to it.

Use the scale below to make your choice:

| **1** | **2** | **3** | **4** | **5** | **6** | **7** |
| --- | --- | --- | --- | --- | --- | --- |
| **never true** | **very seldom true** | **Seldom true** | **Sometimes true** | **Frequently true** | **Almost always true** | **Always true** |

My thoughts cause me distress or emotional pain 1 2 3 4 5 6 7

I get so caught up in my thoughts that I am unable to do the things 1 2 3 4 5 6 7 that I most want to do.

I over-analyse situations to the point where it’s unhelpful to me 1 2 3 4 5 6 7

I struggle with my thoughts 1 2 3 4 5 6 7

I get upset with myself for having certain thoughts

I tend to get very entangled in my thoughts 1 2 3 4 5 6 7

It’s such a struggle to let go of upsetting thoughts even when I know 1 2 3 4 5 6 7

that letting go would be helpful

1. **COM-B Model Questionnaire**

I have the PHYSICAL opportunity to be *vaccinated against COVID-19*.

**What is PHYSICAL opportunity?**

The environment provides the opportunity to engage in the activity concerned.

(e.g. sufficient time, the necessary materials, reminders)

Please rate

Strongly disagree

Strongly Agree

0 1 2 3 4 5 6 7 8 9 10

☐ ☐ ☐ ☐ ☐ ☐ ☐ ☐ ☐ ☐ ☐

I have the SOCIAL opportunity *to vaccinate against COVID-19*.

**What is SOCIAL opportunity?**

Interpersonal influences, social cues and cultural norms provide the opportunity to engage in the activity concerned

(e.g., support from friends and family)

Please rate

Strongly disagree

Strongly Agree

0 1 2 3 4 5 6 7 8 9 10

☐ ☐ ☐ ☐ ☐ ☐ ☐ ☐ ☐ ☐ ☐

I am motivated to *vaccinate against COVID-19*.

**What is motivation?**

Conscious planning and evaluations (beliefs about what is good and bad)

(e.g. I have the desire to, I feel the need to)

Please rate

Strongly disagree

Strongly Agree

0 1 2 3 4 5 6 7 8 9 10

☐ ☐ ☐ ☐ ☐ ☐ ☐ ☐ ☐ ☐ ☐

I am PHYSICALLY able to *vaccinate against COVID-19*.

**What is PHYSICAL capability?**

Having the physical skill, strength or stamina to engage in the activity concerned.

(e.g. I have sufficient physical stamina, I can overcome disability, I have sufficient physical skills)

Strongly disagree

Please rate

Strongly Agree

0 1 2 3 4 5 6 7 8 9 10

☐ ☐ ☐ ☐ ☐ ☐ ☐ ☐ ☐ ☐ ☐

I am PSYCHOLOGICALLY able to *vaccinate against COVID-19*.

**What is PSYCHOLOGICAL capability?**

Knowledge and/or psychological skills, strength or stamina to engage in the necessary thought processes for the activity concerned.

(e.g. having the knowledge, cognitive and interpersonal skills, having the ability to engage in appropriate memory, attention and decision making processes).

Please rate

Strongly Agree

Strongly disagree

0 1 2 3 4 5 6 7 8 9 10

☐ ☐ ☐ ☐ ☐ ☐ ☐ ☐ ☐ ☐ ☐

1. **The Valuing Questionnaire (VQ)**

Please read each statement carefully and then circle the number (0-6) which best describes how much the statement was true for you DURING THE PAST WEEK, INCLUDING TODAY.

1. I spent a lot of time thinking about the past or future, rather than being engaged in activities that mattered to me
2. I was basically on “auto-pilot” most of the time
3. I worked toward my goals even if I don’t feel motivated to
4. I was proud about how I lived my life
5. I made progress in the areas of my life I care most about
6. Difficult thoughts, feelings or memories got in the way of what I really wanted t o
7. I continued to get better at being the kind of person I want to be
8. When things go according to plan, I gave up easily
9. I felt like I had a purpose in life
10. It seemed like I was just ‘going through the motions’, rather than focusing on what was important to me
11. **Socio-demographic information**

Age

Gender:

Male

Female

Other

What is the highest educational level you have completed?

Primary school

High school

Some college/University

Graduated from College/University

Master/Postgraduate studies

Doctoral level

Other

Study programme:

Health Sciences

All other sciences

Do you have children under 18 years of age?

Yes

No

Residence Status:

I live alone

I live with my parents

I live with my own family (partner and/or children)

I live with friends/roommates

1. **COVID-19 infection**

Are you (or have you been) infected with the new coronavirus (COVID-19)?

Yes, I have been diagnosed with COVID-19

No

Don’t know

1. **Institutional trust**

Please rate how much you trust the state authorities.

1=Very little trust, 2, 3, 4, 5, 6, 7=A lot of trust

## Supplementary Material B

*Comparisons between vaccination uptake and intention categories on various included measures (N=484)*

| Measure | Not vaccinated with any dose (*n*=108) | Vaccinated with more than 1 dose but no intention to receive the next dose (*n*=27) | Vaccinated with more than 1 dose but with intention to receive the next dose (*n*=349) | F |
| --- | --- | --- | --- | --- |
|  | Mean (SD) | | |  |
| Institutional Trust | 1.8 (1.2) | 2.2 (1.7) | 4.1 (1.6) | 103.7*^a,b^ |
| Emotional Factors |  |  |  |  |
| Psychological Flexibility | 19.4 (5.4) | 22.4 (7.2) | 22.3 (5.8) | 10.6*^a,b^ |
| Cognitive Factors |  |  |  |  |
| Cognitive Fusion | 23.4 (10.7) | 26.1 (12.7) | 27.5 (10.3) | 6.3*^a,b^ |
| Perceived susceptibility | 7.1 (3.8) | 9.9 (3.6) | 10.7 (3.3) | 45.3*^a,b^ |
| Perceived severity | 7.5 (3.2) | 9.1 (3.7) | 10.8 (3.0) | 46.9*^a,b^ |
| Behavioral Factors |  |  |  |  |
| Values progress | 19.8 (6.8) | 21.3 (6.7) | 19.6 (5.5) | 1.0 |
| Values obstruction | 10.5 (6.4) | 11.6 (8.2) | 12.8 (6.4) | 5.5*^a,b^ |
| Physical Opportunity | 7.4 (3.5) | 8.0 (2.0) | 9.3 (1.5) | 32.3*^a,b^ |
| Social Opportunity | 6.2 (3.6) | 6.6 (2.6) | 9.0 (1.7) | 64.3*^a,b^ |
| Motivation | 2.0 (2.7) | 4.9 (2.9) | 9.0 (1.8) | 484.0*^a,b^ |
| Physical Capability | 7.2 (3.8) | 7.5 (2.8) | 9.7 (.8) | 73.5*^a,b^ |
| Psychological Capability | 3.1 (3.4) | 4.9 (3.7) | 9.0 (1.6) | 297.9*^a,b^ |

*Note.* **p*<.01; ^a^ Post-hoc tests showed significant differences only between participants vaccinated with more than 1 dose but with intention to vaccinate with those being either not vaccinated with any dose or vaccinated with more than 1 dose but no intention to receive the next dose. ^b^ Non-significant differences between those being not vaccinated with any dose with vaccinated with more than 1 dose but no intention to receive the next dose.

## Supplementary Material C

| Determinants | 1 | 2 | 3 | 4 | 5 | 6 | 7 | 8 | 9 | 10 | 11 | 12 | 13 |
| --- | --- | --- | --- | --- | --- | --- | --- | --- | --- | --- | --- | --- | --- |
| 1. Age | - |  |  |  |  |  |  |  |  |  |  |  |  |
| 2. Psychological Flexibility | **-.09*** | - |  |  |  |  |  |  |  |  |  |  |  |
| 3. Cognitive Fusion | **-.20***** | **.39***** | - |  |  |  |  |  |  |  |  |  |  |
| 4. Perceived susceptibility^a^ | **.15**** | **.21***** | **.16***** | - |  |  |  |  |  |  |  |  |  |
| 5. Perceived severity^a^ | **.17***** | **.24***** | **.17***** | - | - |  |  |  |  |  |  |  |  |
| 6. Values progress^a^ | **.12*** | **-.39***** | **-.14**** | -.01 | .01 | - |  |  |  |  |  |  |  |
| 7. Values obstruction^a^ | **-.16***** | **.38***** | **.75***** | **.18***** | **.19***** | - | - |  |  |  |  |  |  |
| 8. Physical Opportunity^a^ | **.14**** | **-.11*** | -.07 | **.19***** | **.20***** | **.10*** | -.04 | - |  |  |  |  |  |
| 9. Social Opportunity^a^ | **.18***** | -.01 | -.01 | **.25***** | **.28***** | .08 | .01 | - | - |  |  |  |  |
| 10. Motivation^a^ | .06 | **.11*** | .08 | **.40***** | **.40***** | -.02 | .06 | - | - | - |  |  |  |
| 11. Physical Capability^a^ | **.10*** | .06 | -.03 | **.23***** | **.26***** | .06 | -.01 | - | - | - | - |  |  |
| 12. Psychological Capability^a^ | .03 | .04 | .02 | **.25***** | **.29***** | -.07 | .04 | - | - | - | - | - |  |
| 13. Institutional Trust | .01 | -.04 | .01 | **.28***** | **.30***** | .08 | .01 | **.33***** | **.41***** | **.62***** | **.33***** | **.53***** | - |

*Correlations between continuous determinants using Pearson’s correlation*

*Note. *p*<.05, ***p*<.01, ****p*<.001.

^a^Correlations between the subscales of the same instrument were not examined.
